# A New Payload Swing Angle Sensing Device and Its Accuracy

**DOI:** 10.3390/s21196612

**Published:** 2021-10-04

**Authors:** Patrik Grosinger, Jan Rybář, Štefan Dunaj, Stanislav Ďuriš, Branislav Hučko

**Affiliations:** Faculty of Mechanical Engineering, Slovak University of Technology in Bratislava, Námestie Slobody 17, 812 31 Bratislava, Slovakia; patrik.grosinger@stuba.sk (P.G.); stefan.dunaj@stuba.sk (Š.D.); stanislav.duris@stuba.sk (S.Ď.); branislav.hucko@stuba.sk (B.H.)

**Keywords:** payload swaying angle sensor, strain gauge, angle measurement, DfAM

## Abstract

Measuring the swing angle of a crane load is a relatively well-known but unsatisfactorily solved problem in technical practice. This measurement is necessary for the automatic stabilization of load swing without human intervention. This article describes a technically simple and new approach to solving this problem. The focus of this work is to determine the accuracy of the measuring device. The focus of this work remains on the design, the principle of operation of the equipment, and the determination of accuracy. The basic idea is to apply the strain gauge on an elastic, easily deformable component that is part of the device. One part of the elastic component is fixedly connected to the frame; the other part is connected to the crane rope by means of pulleys close to the rope. In this way, the bending of the elastic component in proportion to the swing angle of the payload is ensured.

## 1. Introduction

Recently, many state-of-the-art or modern solutions have been introduced even to classic machines such as cranes. One of the most common problems in operating a glow is that it introduces an excessive load sway. To prevent this, control systems are placed onto the electrical part of the crane to help the operator.

A relatively comprehensive overview of the control and measurement systems used to stabilize the crane load swing is given in [1]. Equipment and approaches to control used both in the laboratory and in practice are described here. The approach to the management of this system can be divided into two groups:

- Open-loop control.
- Closed-loop control.

According to [1], the use of fuzzy logic, neural networks, and the optimization of classical control using genetic algorithms are already relatively common approaches to modern control design. A detailed overview of the state-of-the-art control algorithms used to control cranes is also given in [2]. Additionally, in [3], a relatively non-standard estimate of the state vector of the crane is given and is used in the control of the real model. Instead of the usual observations based on the Kalman filter, an analog approach, based on operational amplifiers, is used here. A brief approach to the description and management of this system, which serves as the basis of all the above, is elaborated in [4]. The focus of this publication is not on control algorithms, but on measuring devices used to estimate the swing angle of a load. Therefore, we no longer deal with automatic control.

As far as measuring devices are concerned, the main problem is measuring the swing angle of the load. Measuring the displacements of the trolley and the beam carrying the trolley, as well as measuring the lowering of the hook, rank among simple tasks. For such a measurement, it is possible to use a rotary encoder connected to the rotating parts of the individual units (trolley shift, beam shift, lifting). Such solutions are given in [1,5]. This will not be discussed further as it is a satisfactorily solved problem.

The focus of this article is to measure the swing angle of the load. According to [1], the measuring devices used for measuring the swing angle are divided into contactless and contact-based ones. According to [1], contactless cameras include the use of cameras. The camera or two cameras are mounted onto the trolley. With a sufficiently contrasting background, the applied software recognizes the rope and then it is possible to calculate its angle from perpendicular to the ground. The disadvantages of this approach include the high cost of the cameras and the need for processing software, as well as the need to keep the camera lenses clean. This approach, with the excessively dusty and dirty air, fails. The use of cameras is also applied in [5].

Ref. [5] also lists other solutions to this problem, which are all contact-based methods. Here, a certain part of the rope-load system is connected by some device to the trolley. In [5], the rope is connected to the rotary encoder by means of a fork-shaped device through which the rope passes tightly. When the rope is deflected from the equilibrium position, it carries the fork and rotates it with a rotary encoder. The output of the encoder is then proportional to the rocking angle. This principle is also used in [3,4]. This approach is easy to implement, but its disadvantages include limited accuracy by quantizing the encoder. The encoder has one disc divided into parts. At very small rocking angles, the output of the encoder resembles stairs. Another possibility is to use a gyroscope placed on a crane hook, as mentioned in [6]. In [5], a tilt gauge is used and it is located on the crane hook.

The authors of this article are proposing a new approach to solving this problem. The approach is to use a strain gauge with a highly flexible beam as a sensitive part of the sensor. A utility model has been issued for this device [7]. A brief description of the device with basic measurements is given in [8]. The final prototype of the device was optimized using the Generative Design module in the CAD software Autodesk Fusion 360 [8].

The device proposed in this article is intended for use in connection with feedback control. The device is suitable both for bridge gantry and tower cranes. It is therefore suitable for cranes where the boom is parallel to the ground. It is, however, not suitable for jib cranes that change the angle of the jib to the ground. The construction of cranes from the mechanical point of view has long been standardized and has been discussed in classical publications [9,10].

The primary purpose of a strain gauge is to measure very small displacements. The strain gauge is only a sensing element of the sensor. The whole sensor consists, in addition to the strain gauge itself, of a transducer. The output of the transducer is electrical voltage proportional to the length of the strain gauge. The strain gauge is connected as one of the resistors of the Wheatstone bridge. Changing the ohmic resistance of the strain gauge will cause a difference in the output voltages of the bridge. This voltage difference is very small, thus it needs to be amplified. The Wheatstone Bridge, in most cases, is part of a transducer. The Wheatstone bridge and the principle of its operation in cooperation with strain gauges is also mentioned in modern publications [11,12,13,14]. Too large an electric current must not pass through the strain gauges in the bridge so that they do not heat up and do not change their resistance properties.

An excellent publication deals with the overview of the different types of strain gauges, their use, their development, and their behavior under long-term loading [15]. The use of strain gauges is very diverse today. Recently, numerous new applications of strain gauges have been introduced that are highly beneficial. E.g., in [13] the strain gauges are placed on flexible elements (embedded beams) made of polyester, the deflection of which is proportional to the air flow rate. In this way, the wind speed is measured. The principle of bent beams with strain gauges is also used in our case. In [16], the strain gauges are located in the middle of the cut and subsequently glued as reinforcement into the concrete. The reinforcement with strain gauges embedded in the concrete was then subjected to stress and the stress of the whole was determined. In publication [17], strain gauges are placed on the blade of a figure skate and the magnitude of the acting forces is determined and their influence on various factors is evaluated. In another interesting publication strain gauges are placed in a solidifying epoxy resin in cooperation with thermometers. Today, even extremely small versions of strain gauges are available for various very fine applications, as elaborated in [18,19]. In [18], a miniature strain gauge is placed inside a loaded O-ring seal, in [19] a special strain gauge is designed for placement on human skin. As in our case, strain gauges are used as measuring elements in connection with automatic control, as evidenced by publications [11,12]. Specifically, in connection with the measurement of the angle of rotation, the strain gauge is used as a measuring element in [12]. Progressive methods include the placement of strain gauges in networks, as reported in [20]. In this case, also in cooperation with other sensors, such as thermometers and piezoelectric elements.

New methods of constructing strain gauges are also emerging. In [21] a modern approach to constructing a strain gauge from graphene is proposed. The strain gauge produced this way is flexible and suitable for curved, uneven surfaces and has an extremely fast response.

The first design solutions for strain gauges included resistance wires placed on a stressed body using insulating rollers. Such solutions are no longer used today. A significant advantage of these outdated approaches was minimal measurement hysteresis. There was no foil, or other insulating material, between the examined body and the strain gauge. Subsequently, strain gauges were created, where the resistance wire was wound meanderingly on special paper. The resulting strain gauge was glued to the surface of the stressed body with a specially designed adhesive. This approach is also currently obsolete. Nowadays, foil thin-film strain gauges are mainly used, where the resistance part is formed by screen printing, vacuum evaporation, sputtering, or other methods.

The main factors adversely affecting measurements using strain gauges include sensitivity to transverse stress, transfer of stress from the examined component to the resistive element through the film and adhesive, the influence of ambient temperature and creeping. The adhesive and foil of the strain gauge reduce its sensitivity and causes s delay. The influence of the ambient temperature has a significant negative effect on the strain gauge measurement. The effect of temperature can be so significant that the change in ohmic value caused by temperature reaches values of the magnitude of the change caused by mechanical stress. In order to be able to use strain gauges over a wide temperature range, strain gauges with compensating windings have been developed. There is also a solution using a compensating strain gauge. The compensation strain gauge is located close to the functional strain gauge but is not mechanically stressed. This compensating strain gauge has the same temperature as the functional one and is connected to the Wheatstone bridge in a suitable manner. From the point of view of hysteresis, semiconductor strain gauges are the most preferred. However, their main disadvantage is the considerably nonlinear dependence of the change in resistance on mechanical stress. Semiconductor strain gauges with a high level of additions have a lower temperature dependence but at the expense of sensitivity to mechanical stress. Unlike metal-based strain gauges, these strain gauges have certain photosensitivity. Therefore, protection against lighting is necessary. A detailed overview of strain gauges is given, for example, by a monography [22].

The need to accurately measure the swing angle of the load required the creation of a mechanism with the smallest possible, resp. no wills between the individual elements of the mechanism. Minimal or almost no play can be achieved by very precise machining. This approach is challenging both financially and in terms of the complexity of the whole mechanism. Therefore, a solution has been proposed where flexible elements would be used in the device, and which at first glance would appear as one stationary unit. This approach is extensively described in [23]. When using compliant mechanisms, the DfAM (Design for Additive Manufacturing) method appeared to be more suitable than conventional material machining methods. DfAM gives the designer more freedom in designing, but it also has specific limits that depend on the used additive production technology. The issue is discussed in more detail in [24]. We know several technologies of additive production and each of them requires specific materials. These technologies are constantly evolving, and more of them are added. A basic overview of technologies is given in [25].

Another intention with the design was to optimize the mechanism in terms of shape, rigidity, and weight. DfAM and additive manufacturing allow the designer to use shapes obtained through topological optimization [26] or the Generative design method, which mimics nature’s evolutionary approach to creating structures and objects.

## 2. Device Description

**Figure 1:**
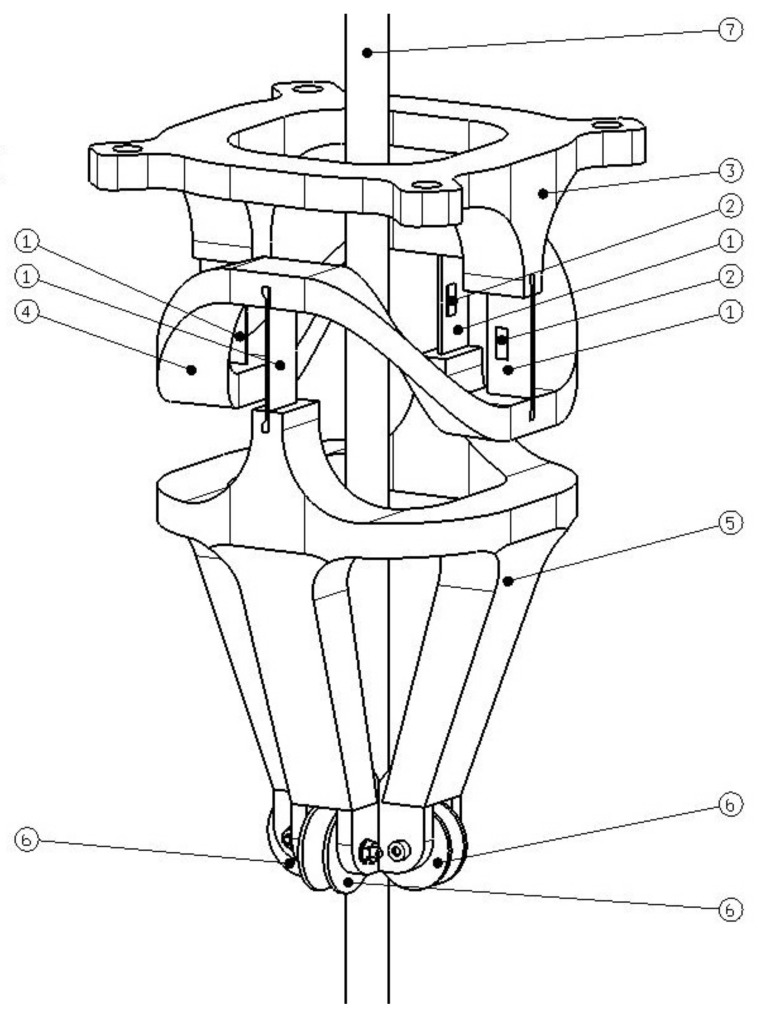
Payload swing angle sensor [7]. (1.) Flexible elements. (2.) Strain gauges. (3.) Device holder mounted on the crane trolley. (4.) An intermediate member performing a spherical motion. (5.) The part of the device performing the movement according to the rope. (6.) Pulleys touching the rope. (7.) Crane rope.

The device designed by us is presented in Figure 1. The aim with this solution was to construct a simple, robust, easy to use, and at the same time sufficiently accurate device for measuring the swing angle. That is, the device can be used as a measuring element in connection with feedback control. The original idea was to use a strain gauge, a component which allows for very small displacements to be measured. First, a simple experiment was performed on an embedded beam made by an additive thermoplastic manufacturing technology. A strain gauge was placed at the inlet where the bend is the largest. When moving the free end of the beam, the strain gauge connected to the transducer provided a voltage output proportional to the deflection of the end, which is proportional to the lengthening or shortening of the beam surface. If one end of the beam was to run parallel to the rope, and the free end of the beam was connected to the rope by means of a guide, the swing angle would correspond to a lengthening or shortening of the strain gauge. The described device is based on these considerations.

The part marked 3 in Figure 1 is firmly connected to the crane cat. This is connected to part 4 by means of a pair of parallel elastic elements 1, on which the strain gauges 2 are placed. Part 4 serves to keep the elastic elements 1 at the same height and to bend each pair only along one of the axes. x or y. Therefore, it is a sort of boundary. Part 5 is connected directly to the rope of the crane 7 by means of pulleys 6. The device designed in this way ultimately represents the attachment of part 5 at one point. Part 5 thus performs a spherical movement in connection with the end of the rope.

**Figure 2:**
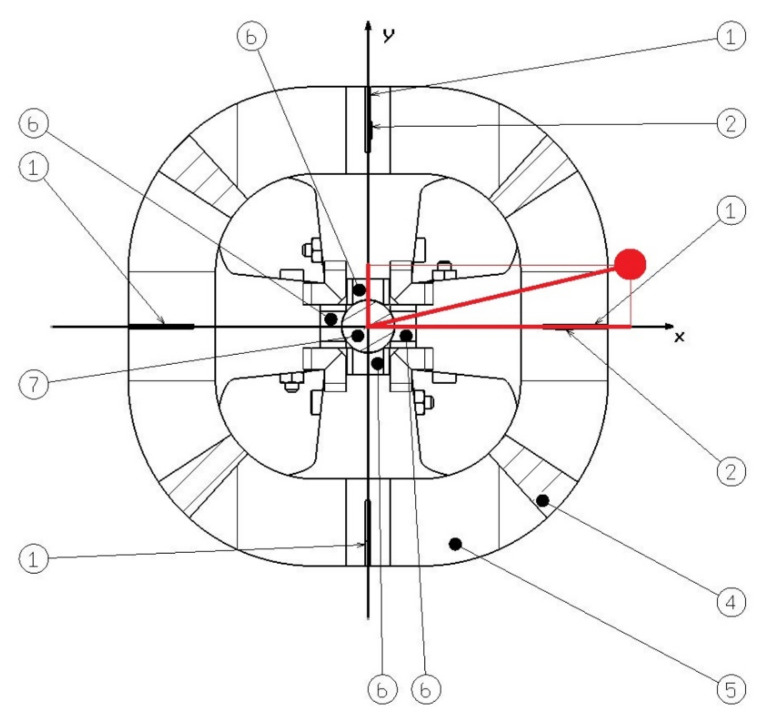
Top view of the device in section through flexible elements (the description of the components is the same as Figure 1).

The operation of the device is clear from Figure 2. If the load moved only in the direction of the x-axis, only the elastic elements lying on the y-axis would bend. The output from the strain gauges would correspond to the swing angle of the load in the plane formed by the x-axis and the rope. Let us denote this angle by $\varphi_{x}$. The load would therefore swing similarly to a simple planar pendulum. Similarly, for the movement of the load in the plane formed by the y-axis and the rope, only the elastic elements lying on the x-axis would be bent. This angle is called $\varphi_{y}$. Let the length of the rope be denoted by l. The angles $\varphi_{x}$ and $\varphi_{y}$ are known on the basis of strain gauges, then we can determine the position of the transformation in space with respect to the x and y axes according to Equation (1).

(1) $$\begin{array}{l} x\left(\varphi_{x},\varphi_{y}\right)=l\sin\varphi_{x}\cos\varphi_{y}\\ y\left(\varphi_{x},\varphi_{y}\right)=l\sin\varphi_{y}\cos\varphi_{x} \end{array}$$

Assuming that the angles $\varphi_{x}$ and $\varphi_{y}$ are small, the relations (1) are simplified to an approximate shape (2).

(2) $$\begin{array}{l} x\left(\varphi_{x}\right)=l\varphi_{x}\\ y\left(\varphi_{y}\right)=l\varphi_{y} \end{array}$$

The entire functional prototype of this device was created using additive manufacturing technologies, thus it was printed on a conventional 3D printer. This approach makes it possible to produce components that cannot be produced in any other way. The parts were very strong and the equipment showed no signs or excessive wear during the tests.

**Figure 3:**
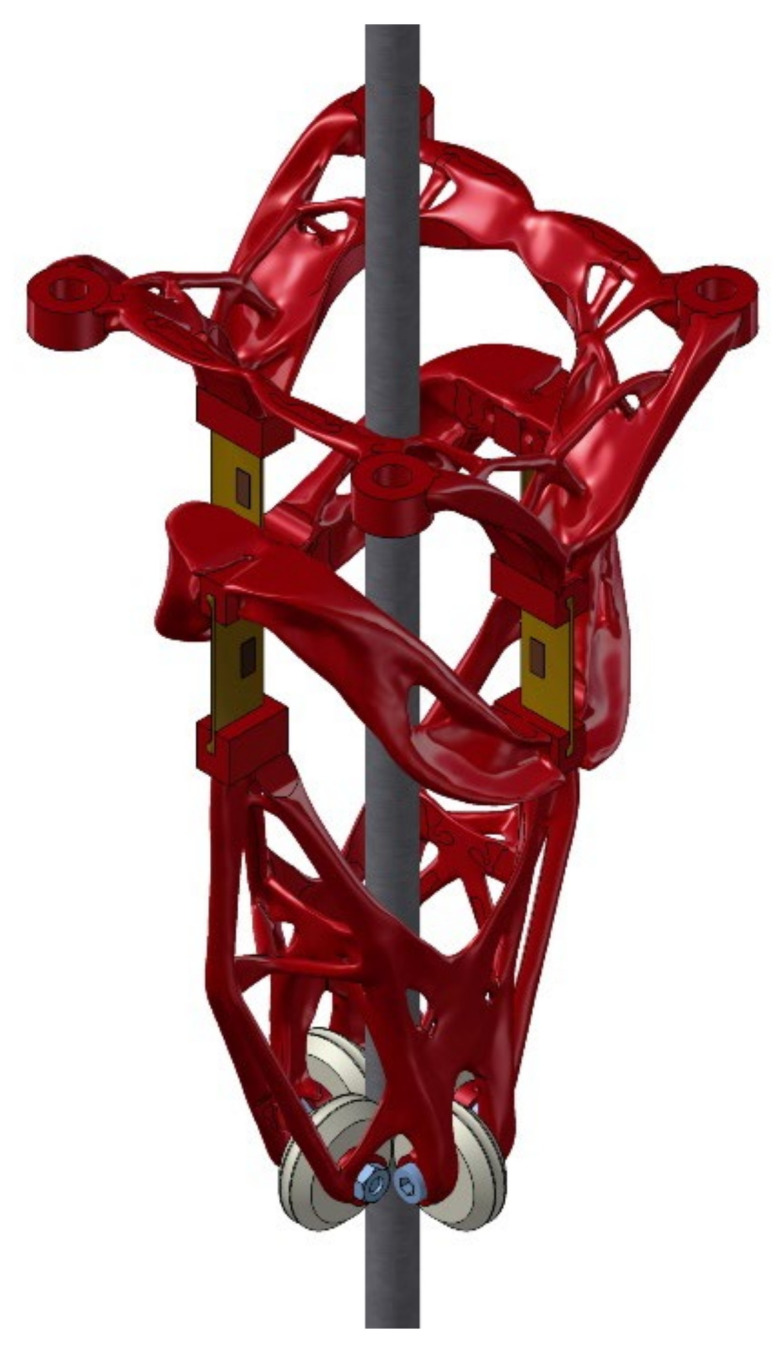
Payload swing angle sensor optimized using a generative design approach.

A more interesting version of the device is in Figure 3. The shape of the device of Figure 1 has been optimized using the Generative Design module so the voltage is the same at each point of the device. Of course, except for the flexible elements with strain gauges, which are the sensitive part of the sensor. At first glance, a highly peculiar shape is, of course, unproducible by conventional chip machining. However, this is not a problem with additive manufacturing technologies. The second prototype of the device was made the same as this. For more information on optimizing the design of this device using the generative design module, see [8]. In this paper, all measurements made were performed on the second prototype shown in Figure 3.

## 3. Description of the Experiment

**Figure 4:**
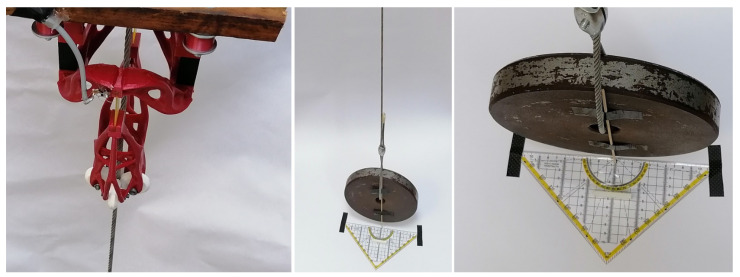
Measurement description: on the left, the sensing device; in the middle and on the right the detail on the ruler and pointer is shown.

The experiment was designed to determine the accuracy and repeatability of the measuring device. In Figure 4, a ruler is visible and a point is attached to the weight on the rope. In the equilibrium position of the system, the tip of the weight points to the zero of the ruler. The ruler has a symmetrical distribution, i.e., from zero in the middle to 10 cm on each side. Thus, it is possible to measure the displacement from the equilibrium position to each side. In this experiment, the pendulum moved only in the plane; the spherical motion would say nothing about the accuracy of the device. Thus, with respect to Figure 2, the ruler placed on the ground is parallel to the x-axis of the measuring device and the load moves in the plane formed by the rope and the x-axis as a planar pendulum.

A total of 10 measurements were performed on the device, always proceeding in one direction from −10 cm to 10 cm on a ruler in 1 cm increments. At the end of each cycle, i.e., the measurement of 21 values belonging to the 21 values on the ruler, the device was allowed to swing freely and then the next measurement was started in the same way. The approach to measurement can be seen in Figure 4. The length of the rope from the attachment to the measuring tip is 196.5 cm. From the value of the rope length, it is then easy to assign an angle in degrees to each distance in centimeters. Centimeters are given in this case because better accuracy could not be achieved by measuring with a ruler and a pointer.

At these dimensions, the maximum swing angle is approximately $\pm 3^{{}^{\circ}}$. In works such as [1,5], larger deviations in sizes of $\pm 5^{{}^{\circ}}$ are also reported. After reducing the gain of the strain gauge transducer, the values of deviation in the range of $\pm 10^{{}^{\circ}}$ were also tested. Fewer measurements were performed, but the nonlinearity of the measuring device did not appear.

The behavior of the measuring device was also not affected by the length of the rope. Experiments with different lengths were tested and the device always measured the angle accurately.

In Table 1, the first column shows the position of the tip on the ruler in centimeters, the second column shows the conversion of this distance to an angle expressed in degrees from perpendicular to the ground. Values in degrees are rounded to two decimal places for clarity, but the full range of computer accuracy was accounted for during processing. In the other columns, named by the serial number of the measurement 1–10, the electrical voltage in volts is indicated, i.e., the output from the transducer for the strain gauge. The transducer was set to give approximately 2.5 V in the middle stable position; after stabilization, the output was 2.454 V. As the angle increases to one side, the voltage increases in proportion to the angle, while it decreases with the other. It should be added that reading the position of the tip from the ruler is certainly not an accurate method of measuring distance. The largest deviations in individual measurements are attributed to this factor. The experiment was performed in a temperature stable environment at 21 °C, therefore errors caused by this factor over time are neglected.

In the experiment, we used a transducer or amplifier designed by us for the strain gauge. Its design is based on the Wheatstone bridge and the instrument’s operational amplifiers. This relatively simple amplifier is characterized by good stability and low noise of the output signal. In this article, no attention will be paid to this device, as for this contribution it is essential to assign a specific voltage value to the corresponding angle.

## 4. Experiment Evaluation

For each angle from Table 1, there are 10 values of the voltage output from the converter. These values were not measured at once, but only after the pendulum was tilted and then returned to the given position. Deviations between these values thus indicate the repeatability of the measuring sensor. Of course, provided that it was read correctly from the ruler and the voltmeter. The voltmeter used was digital, thus there could be no reading error. The statistical analysis methods used were drawn from [27,28].

Before deeper considerations and opinions about the measured data, it is appropriate to test their normality. We will assume that for a single angle from Table 1, all 10 measurements will belong to the normal distribution. In this case, it is advantageous to use the Shapiro–Wilk data normality test. This test requires neither knowledge of $\sigma^{2}$ variance nor knowledge of mean μ. As a null hypothesis, we choose that the data are from the normal distribution of $H_{0}:X∼N\left(\mu ,\sigma^{2}\right)$. As an alternative, we choose the opposite of $H_{1}:X∼!N\left(\mu ,\sigma^{2}\right)$. The α coefficient means the probability of rejecting the null hypothesis, if correct. The measured data are relatively small, thus this coefficient will be chosen as small as possible, i.e., α=0.01. Equation (3) applies to test statistics.

(3) $$W=\frac{{\left(\sum_{i=1}^{m}a_{i}\left(n\right)\left(X_{\left(n-i+1\right)}-X_{\left(i\right)}\right)\right)}^{2}}{\sum_{i=1}^{n}{\left(X_{i}-\bar{X}\right)}^{2}}$$

In relation (3), the $X_{\left(i\right)}$ symbol indicates the ordered random selection variables and the $a_{i}\left(n\right)$ symbol the coefficients corresponding to this test. For the parameter m=n/2 applies, if the number of measurements n is an even number and $m=\left(n-1\right)/2$ otherwise. The rejection of the null hypothesis based on the calculated statistics is decided according to relation (4). Of course, the values of measured stresses for the individual angles were substituted for the variable $X_{i}$.

(4) $$W\leq W_{\alpha }\left(n\right)$$

One case was found where $H_{0}$ was rejected for an angle of 1.46°. Even in this case, the distribution will be considered normal. At other angles, it is unlikely that the normality of the data would be strong and in this case not. The deviation is caused by measurement inaccuracies.

Further results will give the mean values of the measured voltages corresponding to specific angles. These are in Table 2. in the column labeled $\bar{u}$ and their sampling standard errors are s. They are calculated according to Equation (5).

(5) $$\begin{array}{l} \bar{u}=\frac{1}{n}\sum_{i=1}^{n}u_{i}\\ s^{2}=\frac{1}{n-1}\sum_{i=1}^{n}{\left(u_{i}-\bar{u}\right)}^{2} \end{array}$$

In this case, the variance of the individual measurements is not known, thus a normal probability distribution cannot be chosen directly. It is appropriate to choose Student’s normal distribution (t—distribution) of probability, where the variance is unknown. In this case, the variance is estimated from the sample, the so-called sampling variance $s^{2}$.

The accuracy of the measurement with this device will be evidenced by the confidence interval for the mean μ of the normal distribution with unknown variance. For the confidence interval with the specified probability 1−α, relation (6) applies.

(6) $$P\left(\bar{X}-t\left(n-1,\alpha \right)\frac{S}{\sqrt{n}}\leq \mu \leq \bar{X}+t\left(n-1,\alpha \right)\frac{S}{\sqrt{n}}\right)=1-\alpha$$

In (6), $\bar{X}$ is the variable denoting the sample mean and $t\left(n-1,\alpha \right)$ is the coefficient for Student’s distribution depending on the parameters in parentheses. Using (6), Table 2 was calculated, where $l_{95}$ is the lower limit of the confidence interval with a probability of 95% and $h_{95}$ the upper limit for the same probability. Similarly, the limits for 99% probability are indicated in Table 2. The confidence intervals found are so narrow that they cannot be clearly shown in the graph. For this reason, they are given in the form of a table. This indicates a relatively high accuracy of the measuring device.

**Figure 5:**
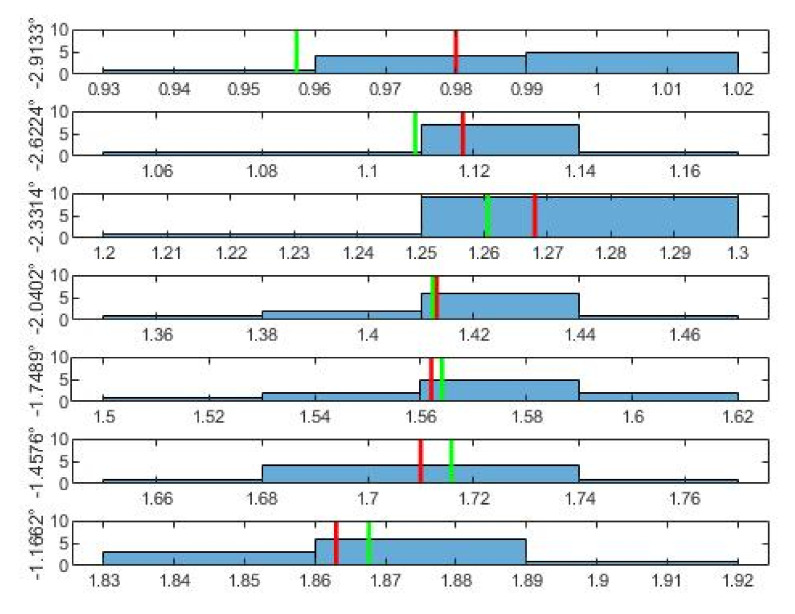
Histograms of the first 7 measurements. On the left is the appropriate angle and multitude. The red line is the mean $\bar{u}$; the green line is the LSM value of u appropriate to the angle.

**Figure 6:**
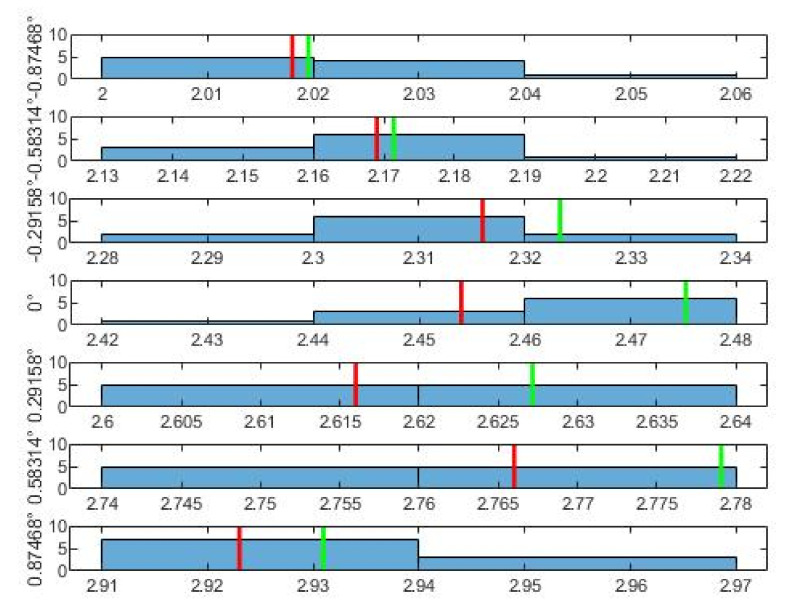
Histograms of the second 7 measurements. On the left there is the appropriate angle and multitude. The red line is the mean $\bar{u}$; the green line is the LSM value of u appropriate to angle.

**Figure 7:**
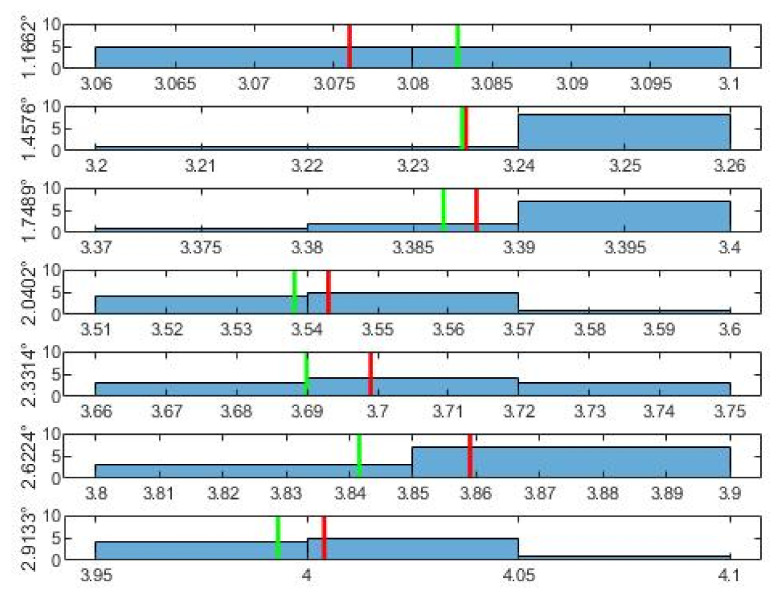
Histograms of the third 7 measurements. On the left there is the appropriate angle and multitude. The red line is the mean $\bar{u}$; th green line is the LSM value of u appropriate to angle.

As for the normal distribution of the data belonging to each angle, these are shown by the histograms in Figure 5, Figure 6 and Figure 7. From the histograms, a distant form with a normal distribution is visible. The red vertical line shows the mean value of the measurements for a specific angle $\bar{u}$ from Table 2. The green line shows the value that results for the given angle from the least squares method. The least squares method was used to obtain an approximation of the dependence between the angle $\varphi^{{}^{\circ}}$ as the input, and between the mean values of the voltage $\bar{u}$ as the output. From the least squares method, the final conversion relationship for the measuring device was obtained (7).

(7) $$u=0.5210\varphi^{{}^{\circ}}+2.4752$$

**Figure 8:**
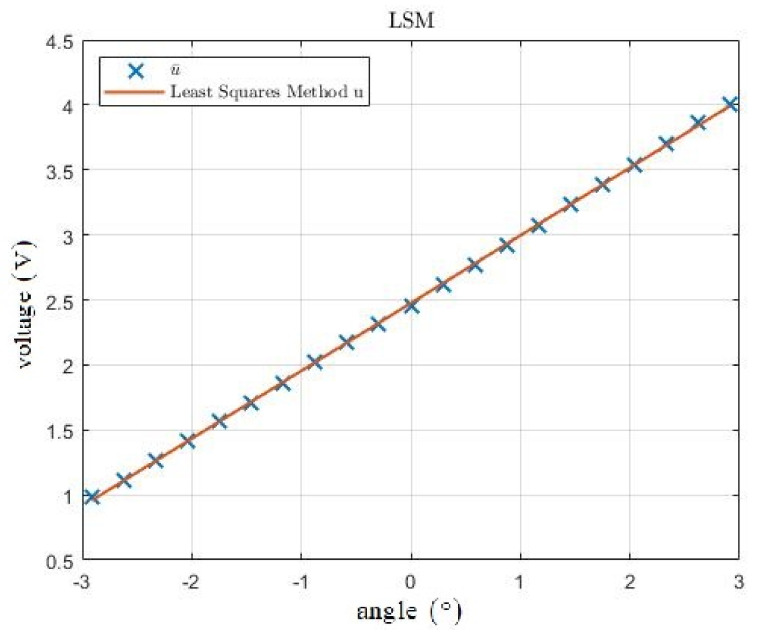
Comparison between means $\bar{u}$ of measured data (blue crosses) and LSM approximation (red line).

**Figure 9:**
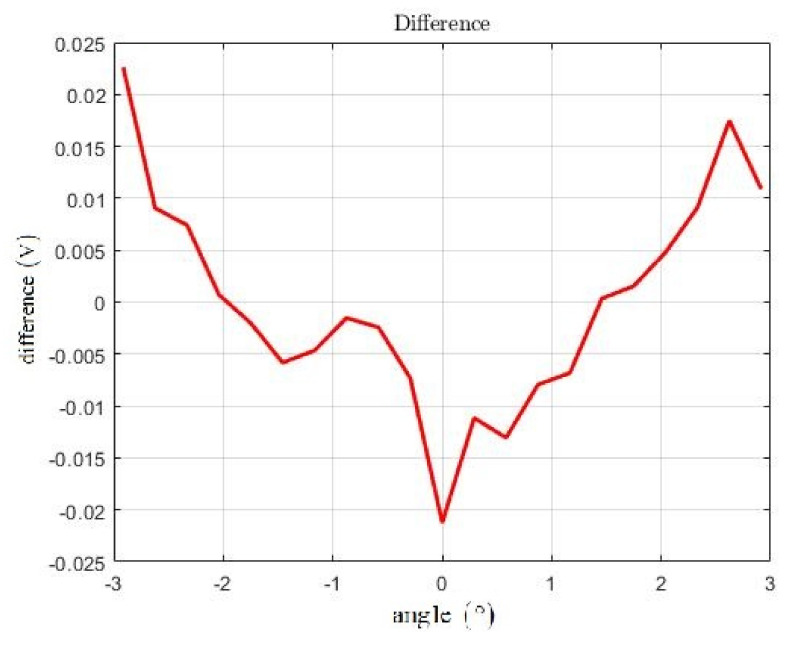
Comparison between means $\bar{u}$ of measured data (blue crosses) and LSM approximation (red line).

The processing of the measured data is shown in Figure 8. The blue crosses indicate the $\bar{u}$ values assigned to the individual angles; the red line is a representation of function (7) obtained by the least squares method. The line with a particularly good agreement is visible and at first glance the data have a strongly linear dependence. The deviations between the $\bar{u}$ values and function (7) are shown in Figure 9. The maximum deviation is approximately 0.023 V.

We will consider the equation of a line in the form of y=kx+q. If the least squares method is used for each of the measurements in 1 to 10 of Table 1, a pair of $k_{i}$ and $q_{i}$ parameters are obtained for each measurement. These are listed in Table 3. The significance of this table lies in the comparison of $k_{i}$ and $q_{i}$ values, which indicate differences in the individual measurements. According to the comparison of the individual coefficients, considerable similarity between individual measurements can be seen. For a better evaluation, it is appropriate to perform a test of data normality according to Shapiro–Wilk. As in the previous case, we consider the α=0.01 coefficient. For both selections $k_{i}$ and $q_{i}$, the hypothesis $H_{0}$ is accepted, thus their distribution can be considered normal. Then, the values given in Table 4 give a clear picture of the selection.

**Figure 10:**
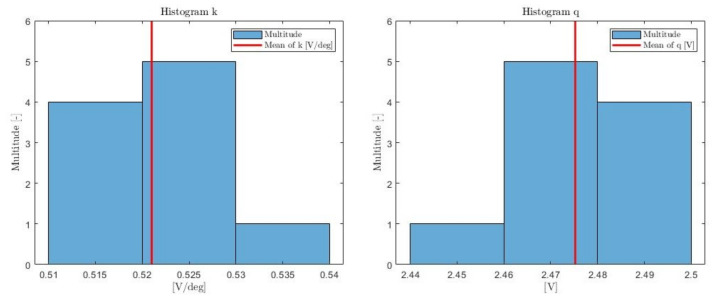
Histogram of k and q parameters.

The histograms for the $k_{i}$ and $q_{i}$ selections are visible in Figure 10. The histograms show a clear distribution. The red line indicates the sampling average of the data from Table 3. The considerable accuracy of the measuring device is proved mainly by Table 4. Sampling variances and sampling standard errors acquire extremely small values.

However, when using this angle measuring device, the opposite relation to the relation (7) is required. It is necessary to calculate the measured angle from the voltage output. A simple calculation arrives at the relation (8).

(8) $$\varphi^{{}^{\circ}}=1.9194u-4.7509$$

A ProsKit MT-1705 digital multimeter was used to measure the electrical voltage. The course of the output voltage during the swing of the load was monitored by a Peak-Tech oscilloscope. All data processing was performed using MATLAB.

## 5. Discussion

The aim of the proposed device is to enable crane control in negative feedback. In this case, the biggest problem in technical practice is the measurement of the swing angle of the load. The results obtained indicate that this device is suitable for the purpose and is sufficiently accurate.

It is worth mentioning the so-called string effect, a process that takes place independently of the swing of a payload. The rope very often oscillates similarly to a string and generates vibrations. However, these vibrations have a significantly higher frequency spectrum than the frequency spectrum of the swinging load. They can therefore be easily filtered out. There is also some noise caused by the rope passing through the pulleys of the measuring device when lifting and lowering the load. The surface of the rope is not perfectly smooth. The swing angle measured in this way is also enriched with undesired components which have nothing to do with the swing angle. It has been found experimentally that these components are significantly higher in frequency than the frequency of the load swing.

Thus, the disadvantage of this approach is that the device also measures undesirable components of the crane-load system. It therefore measures the components that the standard dynamic model (two-mass system) ignores. Thus, a suitably adjusted filtration of higher harmonic components of the measured signal is required. However, all standard measurement methods mentioned in the introduction also have this disadvantage. The transfer function of the strain gauge itself, which already has an integrative character at relatively low frequencies, contributes in part to the elimination of this problem. It therefore behaves naturally as a filter. According to experiments with the device, not directly related to the content of this article, but filtering of the measured signal is necessary.

When using this device, compared with the use of stereo cameras, there is no need to use powerful and expensive hardware and software. The results are comparable and the possibility of an error caused by spillage is eliminated. Compared with the use of gyroscopes and accelerometers, there is no need for relatively complex filtration and other data processing, as required by different versions of the Kalman filter. For example, when using a standard Kalman filter, knowledge of the system dynamics is required and is constantly changing in the case of a crane. Using the sensing device we designed does not create the need to know the dynamics of the system and the angle measurement gives usable results for feedback control.

## 6. Conclusions

Compared with previous approaches, this approach has several advantages. The measuring device is easy to manufacture and therefore inexpensive. Additionally, according to the results above, it is highly accurate. The device is in the prototype phase. Additionally, for mounting on a real machine, it would be necessary to structurally modify the device so that it is detachable. In the state shown in the figures, it is necessary to thread the rope through the measuring device, which is disadvantageous and unacceptable outside the laboratory. However, this problem is extremely easy to solve. The use of additive manufacturing technologies is also considered for the next prototype, which is placed on a real crane.

The accuracy of the device is sufficient for the purposes of automatic control, as determined by statistical methods. The deviations between the measured and the real swing angle are negligible. The measured and subsequently evaluated data correspond to the theoretical side in the use of strain gauges in a practical mechatronic application, the device can be used properly for the described purposes in technical practice.

## Figures and Tables

**Table 1:** Measured data.

Displacement (cm)	Angle φ (°)	1 (V)	2 (V)	3 (V)	4 (V)	5 (V)	6 (V)	7 (V)	8 (V)	9 (V)	10 (V)
−10	−2.91	0.98	0.97	0.99	0.94	0.98	0.99	0.99	0.97	1.00	0.99
−9	−2.62	1.12	1.11	1.14	1.07	1.09	1.12	1.13	1.13	1.15	1.12
−8	−2.33	1.27	1.26	1.28	1.21	1.27	1.26	1.30	1.27	1.30	1.26
−7	−2.04	1.43	1.40	1.44	1.36	1.42	1.40	1.43	1.41	1.43	1.41
−6	−1.75	1.59	1.56	1.59	1.52	1.56	1.55	1.57	1.56	1.57	1.55
−5	−1.46	1.74	1.70	1.73	1.67	1.70	1.70	1.72	1.72	1.72	1.70
−4	−1.17	1.89	1.86	1.88	1.83	1.85	1.85	1.87	1.86	1.88	1.86
−3	−0.87	2.04	2.01	2.03	2.00	2.00	2.01	2.03	2.02	2.03	2.01
−2	−0.58	2.20	2.17	2.18	2.15	2.15	2.16	2.18	2.18	2.17	2.15
−1	−0.29	2.32	2.33	2.32	2.30	2.30	2.31	2.32	2.32	2.33	2.31
0	0.00	2.43	2.46	2.46	2.46	2.44	2.45	2.47	2.46	2.47	2.44
1	0.29	2.62	2.61	2.61	2.61	2.60	2.61	2.63	2.62	2.63	2.62
2	0.58	2.76	2.77	2.79	2.76	2.75	2.76	2.78	2.77	2.78	2.77
3	0.87	2.91	2.92	2.91	2.95	2.91	2.91	2.93	2.94	2.94	2.91
4	1.17	3.06	3.09	3.07	3.09	3.06	3.07	3.08	3.09	3.09	3.06
5	1.46	3.24	3.24	3.22	3.24	3.20	3.24	3.25	3.24	3.24	3.24
6	1.75	3.39	3.40	3.38	3.39	3.37	3.38	3.39	3.40	3.39	3.39
7	2.04	3.56	3.58	3.51	3.55	3.51	3.53	3.55	3.56	3.55	3.53
8	2.33	3.73	3.72	3.67	3.71	3.66	3.68	3.69	3.72	3.70	3.71
9	2.62	3.90	3.88	3.82	3.87	3.81	3.89	3.83	3.87	3.85	3.87
10	2.91	4.08	4.03	3.97	4.04	3.96	3.98	3.98	4.00	4.00	4.01

**Table 2:** Totals of 99% and 95% confidence intervals, Student’s probability distribution.

Angle φ (°)	$l_{99}$ (V)	$l_{95}$ (V)	$\bar{u}$ (V)	$h_{95}$ (V)	$h_{99}$ (V)	s (V)
−2.91	0.9625	0.9678	0.980	0.9922	0.9975	0.0170
−2.62	1.0939	1.1012	1.118	1.1348	1.1421	0.0235
−2.33	1.2420	1.2499	1.268	1.2861	1.2940	0.0253
−2.04	1.3892	1.3965	1.413	1.4295	1.4368	0.0231
−1.75	1.5410	1.5474	1.562	1.5766	1.5830	0.0204
−1.46	1.6894	1.6957	1.71	1.7243	1.7306	0.0200
−1.17	1.8448	1.8504	1.863	1.8756	1.8812	0.0177
−0.87	2.0036	2.0080	2.018	2.0280	2.0324	0.0140
−0.58	2.1519	2.1571	2.169	2.1809	2.1861	0.0166
−0.29	2.3050	2.3083	2.316	2.3237	2.3270	0.0107
0.00	2.4401	2.4443	2.454	2.4637	2.4679	0.0135
0.29	2.6061	2.6091	2.616	2.6229	2.6259	0.0097
0.58	2.7561	2.7591	2.766	2.7729	2.7759	0.0097
0.87	2.9069	2.9118	2.923	2.9342	2.9391	0.0157
1.17	3.0621	3.0663	3.076	3.0857	3.0899	0.0135
1.46	3.2203	3.2247	3.235	3.2453	3.2497	0.0143
1.75	3.3786	3.3814	3.388	3.3946	3.3974	0.0092
2.04	3.5197	3.5268	3.543	3.5592	3.5663	0.0226
2.33	3.6750	3.6823	3.699	3.7157	3.7230	0.0233
2.62	3.8278	3.8373	3.859	3.8807	3.8902	0.0303
2.91	3.9664	3.9778	4.004	4.0302	4.0416	0.0366

**Table 3:** Means of k and q parameters for each measurement.

	1	2	3	4	5	6	7	8	9	10
$k_{i}$ (V/°)	0.5242	0.5274	0.5106	0.5349	0.5145	0.5208	0.5160	0.5232	0.5167	0.5218
$q_{i}$ (V)	2.4886	2.4795	2.4743	2.4629	2.4567	2.4690	2.4819	2.4814	2.4867	2.4714

**Table 4:** Selection variances and errors of k and q parameters.

	$s^{2}$	s
k	$1.7270\times 10^{-16}$	$1.3142\times 10^{-8}$
q	$1.0683\times 10^{-4}$	0.0103

## Data Availability

The data are given in the text of the article.
